# Amphetamine-induced prolonged disturbances in tissue levels of dopamine and serotonin in the rat brain

**DOI:** 10.1007/s43440-023-00472-6

**Published:** 2023-03-21

**Authors:** Ewa Taracha, Magdalena Czarna, Danuta Turzyńska, Piotr Maciejak

**Affiliations:** 1 Department of Neurochemistry, Institute of Psychiatry and Neurology, 9 Sobieskiego St., 02-957 Warsaw, Poland; 2 Department of Experimental Oncology and Preclinical Research, The Maria Sklodowska-Curie National Research Institute of Oncology, 5 Wilhelma Roentgena St., 02-781 Warsaw, Poland; 3 Department of Experimental and Clinical Pharmacology, Centre for Preclinical Research and Technology, Medical University of Warsaw, 1B Banacha St., 02-097 Warsaw, Poland

**Keywords:** Long-term withdrawal, Dopamine–serotonin interaction, Addiction, Psychostimulants ultrasonic vocalization

## Abstract

**Background:**

A hallmark of psychostimulants is the persistence of neurobiological changes they produce. The difficulty in reversing long-time effects of psychostimulants use is why addiction therapy is so ineffective. This study aimed to look for such drug-induced changes that can be detected even after many weeks of abstinence.

**Methods:**

Rats were given 12 doses of amphetamine (Amph) at 1.5 mg/kg. The rewarding effect of Amph was assessed using ultrasonic vocalization. After 14 and 28 days of abstinence, tissue levels of dopamine (DA), serotonin (5-HT), and their metabolites were measured in the prefrontal cortex (PFC), nucleus accumbens (Acb), dorsomedial (CPuM), and dorsolateral (CPuL) striatum.

**Results:**

After 28 days of abstinence, DA levels were increased in the dorsal striatum while 5-HT levels were decreased in all brain regions studied. The opposite direction of changes in DA and 5-HT tissue levels observed in the dorsal striatum may be related to the changes in the emotional state during abstinence and may contribute to the incubation of craving and relapses. Tissue levels of 5-HT and DA showed intra- and inter-structural correlations, most pronounced after 14 days of abstinence. Most of them were absent in the control group (ctrl), which may indicate that their appearance was related to the changes induced by earlier Amph administration. We did not find any associations between reward sensitivity and the persistence of Amph-induced neurochemical disturbances.

**Conclusions:**

Administration of 12 moderate doses of Amph causes prolonged changes in DA and 5-HT tissue levels. The direction and severity of the changes are dependent on the brain region and the neurotransmitter studied.

## Introduction

The abuse of stimulants such as amphetamine (Amph) and its substituted derivatives is increasing worldwide, especially in Asia. Globally, the number of people requiring treatment for this type of addiction is also increasing [1]. Since no approved drug therapy is available and no effective treatment has been proposed yet, further research in this field is needed. When taken in relatively low doses, Amph results in improved mood, experiences of euphoria, increased alertness, feeling of confidence, and suppression of fatigue. In contrast, regular and prolonged use of Amph and its substituted derivatives harms physical status and increases the risk of psychiatric disorders, including psychosis and depression, and the onset of suicidal attempts and aggressive behavior [2].

Addiction is a complex phenomenon, its emergence is determined by various factors. Depending on the problem studied, different experimental models are used. Since it is not possible to mimic all the symptoms of Amph addiction with a single animal model, several self-administration schemes are used to study the reinforcing effects of drugs, pathological learning, or motivation [3]. The persistence of drug-induced changes may contribute to relapse even after a long period of abstinence [4]. To study this problem, an experimental paradigm in which neurochemical measurements are made long after the drug is expelled from the body, has been proposed.

Since almost all addictive substances are rewarding and affect the reward, motivation, and memory circuits [5], in the present study the rewarding effects of Amph were examined. For this purpose, rodent studies often use ultrasonic vocalization (USV), a common mode of communication in these species [6, 7]. Vocalization in the appetitive band, especially the frequency-modulated form of the 50-kHz FM USV, is usually associated with positive affective states elicited by (or in anticipation of) naturally rewarding events, such as access to food and social or sexual interaction [8]. It can also be induced by addictive substances, e.g., cocaine [9] or Amph [10], or by the drug context [11]. In addition, FM 50-kHz USV shows a high inter-individual, but a low intra-individual variability [12], which suggests the possibility of using 50-kHz USV as an indicator of the sensitivity of individual animals to rewarding effects. It has been shown that 50-kHz USV can reflect a range of typical effects of addictive agents, such as sensitization and tolerance which predisposes it to monitor their behavioral effects. A further argument for using 50-kHz USV is that it requires activation of the mesolimbic dopamine system [13, 14]. The rewarding properties of many addictive substances have been linked to ascending dopaminergic pathways, including nigrostriatal, mesocortical, and mesoaccumbal systems. DA is important for the psychostimulant rewarding effect following acute administration and also for the permanent neuroadaptive changes in the brain accountable for the ingrained behaviors characteristic of drug addiction [15, 16].

Serotonin (5-HT), a neurotransmitter essential for maintaining neuroplasticity throughout the lifespan [17], involved in motivation and reinforcement processes as well as learning and memory associated with drug use behavior [18–20], also plays a significant role in the development of addiction. The value and the motivational aspects of rewards induced by, for example, intracranial self-stimulation are mediated by serotonergic innervation of the limbic-corticostriatal circuit [21, 22]. Dopaminergic brain regions, including the substantia nigra and ventral tegmental area, and their terminal fields are innervated by the 5-HT system [23, 24]. This anatomical proximity of DA and 5-HT neurons points to a potential role for 5-HT in regulating DA network activity. This has brought to attention the relevance of the serotonergic system in controlling drug use behavior, the transition to compulsive use, and the emergence of addiction, in which the dopaminergic system plays a critical role. It has been shown that increase or decrease in 5-HT neurotransmission can decrease or increase, respectively, the potential of Amph and cocaine to induce a dopaminergic response [25, 26].

Interactions between 5-HT and DA are complex; their effects are mediated by neuronal circuits involving also other transmitters. Some effects evoked by drugs, such as the so-called "high" or the DA or 5-HT release, are short-term but other permanent effects can also occur. Even a single drug dose can produce long-term behavioral and neurochemical effects [27, 28], resulting in relapses even after a prolonged period of abstinence. Most studies focus on the rapidly changing, transient effects of psychostimulants, such as the release of neurotransmitters in response to acute Amph administration [29–31]. The researchers also studied the response to stimuli, such as priming and cues in detoxified individuals, or investigated the effects shortly after the end of self-administration [32–34]. On the other hand, few studies address changes that remain long after drugs are eliminated from the body.

The longevity of drug-induced changes is essential to the development of addiction and is responsible for the difficulty in developing effective therapy. Therefore, this work aimed to study the changes in tissue DA and 5-HT levels and interactions between them in the brain structures crucial for the development of addiction that persist for many weeks after the Amph withdrawal.

## Materials and methods

### Rats

For the study, 36 male Sprague–Dawley rats were selected from the Mossakowski Medical Research Center stock in Poland. The initial body mass of the animals was approximately 300 g. Rats were housed individually in transparent plastic cages in a local animal facility. Conditions in the animal room were controlled (20–22 °C and 45–65% relative humidity), with a 12 h/12 h light/dark cycle (light on at 7 am). The animals were housed individually to ensure optimal experimental conditions, as this research refers to previous studies in which such conditions were necessary [36]. Throughout the trial, rats were given free access to food (Morawski Animal Feeds Factory, Kcynia, Poland; http://www.wpmorawski.com.pl) and tap water. All procedures used in the experiments were performed between 7 am and 7 pm, during the light period. All reasonable efforts were made to minimize animal suffering and the number of rats used. All animal use protocols complied with current Polish law and the European Parliament and Council Directive 2010/63/EU on the protection of animals used for scientific purposes, which was adopted on September 22, 2010. The Second Local Ethical Committee for Animal Experimentation, Life Sciences University of Warsaw, Warsaw, Poland, approved the study protocol. (Permit No. WAW2/042/2018).

### Drugs

Rats were given intraperitoneal injections (*ip*) of d-amphetamine sulfate (Catalog number A634295, Toronto Research Chemicals, Canada) dissolved in sterile 0.9% NaCl solution in deionized water at 1.5 mg/ml. Amph doses are expressed as the weight of the salt.

### Experimental design

**Fig. 1:**
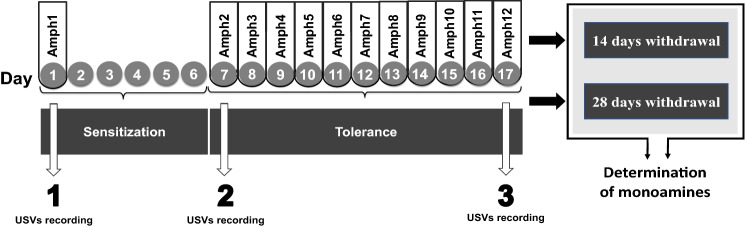
General scheme of experiment. Amph1–Amph12 indicates consecutive intraperitoneal injections of Amph (1.5 mg/kg). The control group received NaCl. USV was recorded for Amph1, Amph2 and Amph12. *Amph* amphetamine

Rats received 12 doses of Amph (test group) or 0.9% NaCl solution (ctrl; control group) (see Fig. 1) using the TIPS (Two Injection Protocol of Sensitization) procedure [35] previously used in our laboratory [37]. According to this procedure, the first dose (Amph1) induces sensitization seen in response to the 2nd dose (Amph2) given after the time required for the biological changes (Day 7) induced by Amph1 to occur. In this study, Amph2 was also the first in a series of 11 daily doses of the drug (Amph2-Amph12) designed to induce adaptive processes, such as tolerance. Frequency-modulated ultrasonic vocalization (FM 50-kHz USV) was used to monitor the behavioral effects of Amph [9, 13, 14, 38] on Days 1 (Amph1 effect), 7 (sensitization), and 17 (final effect of Amph administration). Since the behavioral effect of psychoactive substances depends on whether they are administered in home cages or a drug-specific environment [39, 40], measurements were carried out in dedicated laboratory rooms in non-home cages separated by soundproof walls. USV was recorded at two-time intervals: First, it was recorded 20 min before injection. Measurement prior to Amph1 assessed the spontaneous vocalization of naïve rats. Subsequent measurements before Amph2 and Amph12 assessed the response to the environment/context of Amph administration. This 20 min before injection allowed for the extinction of responses other than the response to Amph administration [41]. Rats were then injected intraperitoneally with Amph or 0.9% NaCl, and USV was recorded for another 20 min. After the experiment, the animals were returned to their home cages. Daily doses of Amph on the remaining non-measured days (Amph3–Amph 11) were also administered in the measurement rooms, following the same procedure as during the measurements. The experiment involved two test groups plus a control group (*n* = 8), for a total of 36 rats. Both test groups received Amph according to the schedule (see Fig. 1), but one group had 14 days of withdrawal (*n* = 14) and the other group had 28 days of withdrawal (*n* = 14). Accordingly, 14 and 28 days after the last dose, the rats were decapitated, and their brains were removed from the skull, frozen in isopentane (− 76 °C), and stored at − 76 °C until analyzed for dopamine, DOPAC, HVA, 5-HT, and 5-HIAA using HPLC. Using tissue punches with an internal diameter of 1.8 mm (Biovivet 2.1 × 60 mm veterinary needles), tissue was collected from the following brain areas: the prefrontal cortex (coordinates: A: 5. 16), Acb (coordinates: A: 2. 28, L: 1.5, V: 7.0), CPuM (coordinates: A: 0.6, L:2.4, V: 4.5) and CPuL (coordinates: A: 0.6, L:3.8, V: 4.5) according to the rat brain atlas by Paxinos and Watson.

### USVs recording

The recording equipment and techniques for all USVs were described previously by Taracha et al. [37]. Briefly, USV calls were collected with a single model CM16 condenser microphone (Avisoft Bioacoustics, Germany) from each rat. The microphones were sensitive to frequencies of 15–180 kHz and were coupled with a custom-made amplifier of 600 Ω input impedance, 16 V/V (12 dB) voltage gain, and ± 0.1 dB (30 Hz–100 kHz) frequency response. The amplified signal was passed through a custom-made anti-aliasing filter and then transferred to a PC equipped with a PCI-703-16A acquisition board (Eagle Technology, Eagle River, WI, USA) and a custom-written software (Rat-Rec Pro 5.0), processed by a fast Fourier transform and displayed as a color spectrogram. According to Brudzynski et al. [6], non-FM ('flat') and FM 50-kHz USVs were identified; the latter category includes all calls with frequency variation > 5 kHz. Flat calls accounted for just a small percentage (1–2%) of all 50-kHz USV sounds in our research (Taracha et al. unpublished data). Hence only FM 50-kHz USVs were examined. During this study, no aversive (22 kHz) calls were identified.

### Determination of monoamines and their metabolites

Each tissue sample was weighed, placed in a dry-cooled polypropylene vial, and homogenized in 20 volumes of ice-cold 2 percent perchloric acid with dihydroxybenzylamine as the internal standard for 30 s at 4 °C, using a VirSonic 50 ultrasonic cell disrupter set at 25% output power for the determination of monoamines and their metabolites. The homogenates were centrifuged at 26.880*g* for 8 min. at 4 °C. The supernatants were collected after centrifugation and filtered through a 0.45 m filter (Millipore). The sample was instantly frozen and maintained at − 76 °C until it was analyzed. Using a modified high-pressure liquid chromatography (HPLC) method previously published by Kaneda et al. [42], the levels of monoamines and their metabolites were measured. The Shimadzu UFLC LC-20ADvp pump and an electrochemical detector with a flow-through cell (Waters 2465) made up the HPLC system. At + 800 mV, a high-density, glassy carbon-working electrode was used.

The sample was manually injected using a 10 µl sample loop and a Rheodyne 7725i injection valve. On a Phenomenex—Kinetex C18 100A (150 mm × 4.6 mm, 5 micron particle size) with a Phenomenex AJO-9000 precolumn, the separation of monoamines and their metabolites was accomplished. The temperature of the column was 32 °C. The mobile phase was filtered using 0.45 µm filters (Millipore) and included 64.4 mM disodium phosphate (Na_2_HPO_4_), 67.8 mM citric acid (C_6_H_8_O_7_), 0.054 mM EDTA, 0.387 mM octane sulfonic acid (C_8_H_17_NaSO_4_), 2.0 mM potassium chloride (KCl), and 12% methanol. The flow rate was 1.0 ml/min. and the mobile phase was degassed with helium. The Chro-mod 2007 software (Pol-Lab) program was used to register and analyze the chromatograph. The amounts of dopamine (DA), 3,4-Dihydroxyphenylacetic acid (DOPAC), homovanillic acid (HVA),serotonin (5-HT), 5-Hydroxyindoleacetic acid (5-HIAA) in the brain tissue were estimated in nmol/g tissue.

### Statistics

All data were expressed as the mean + SEM. The neurochemical analysis data were subjected to a one-way analysis of variance (ANOVA), followed by post hoc Tukey’s test. Since the USVs data showed significant variance heterogeneity and non-normal distribution, they were square root-transformed for statistical analyses [37]. The transformed USVs and rats’ body mass data were subjected to a two-way repeated measures ANOVA followed by the Tukey test.

The intra-structural and inter-structural correlation coefficients between individual neurochemical levels for single groups were calculated using Pearson’s *r* correlation analysis. To keep the probability of the family-wise type I error rate < 0.05 the sequential Bonferroni–Holm correction was applied to the results of the analyses when appropriate [43]. In all cases, a *p* < 0.05 was considered significant. All the analyses were run with the Statistica v. 13.3 software package (StatSoft, Tulsa, OK, USA).

## Results

### Effect of repeated amphetamine treatment on the body mass of rats

The body mass of the rats in the ctrl and the group receiving Amph changed similarly throughout the experiment. Two-way repeated measures ANOVA revealed a consecutive injections effect (*F*_2,68_ = 218.90, *p* < 0.001) but no treatment effect (*F*_1,34_ = 1.29, *p* = 0.26) and treatment × consecutive injections interaction effect (*F*_2,68_ = 1.54, *p* = 0.22). The average body mass of rats in the ctrl at the first injection was 289 ± 11.12 g, while that for the test group was 260 ± 9.36 g. At the last injection of NaCl or Amph, the average body mass for the ctrl was 386 ± 17.50 g and that for the test group was 375 ± 6.71 g (Table 1).

**Table 1:** Body mass of animals during multiple administrations of Amph (*n* = 28) and NaCl (*n* = 8)

		Body mass (g)		
		Day 1	Day 10	Day 17
Treatment	NaCl	289	373	386
	Amph	260	358	375

Day 1 corresponds to the first injection, day 10 to the fifth injection, and day 17 to the last (12th) injection. *Amph* amphetamine

### Effects of repeated amphetamine treatment on the FM 50-kHz USV rate

#### Context-induced FM 50-kHz USV rate

**Fig. 2:**
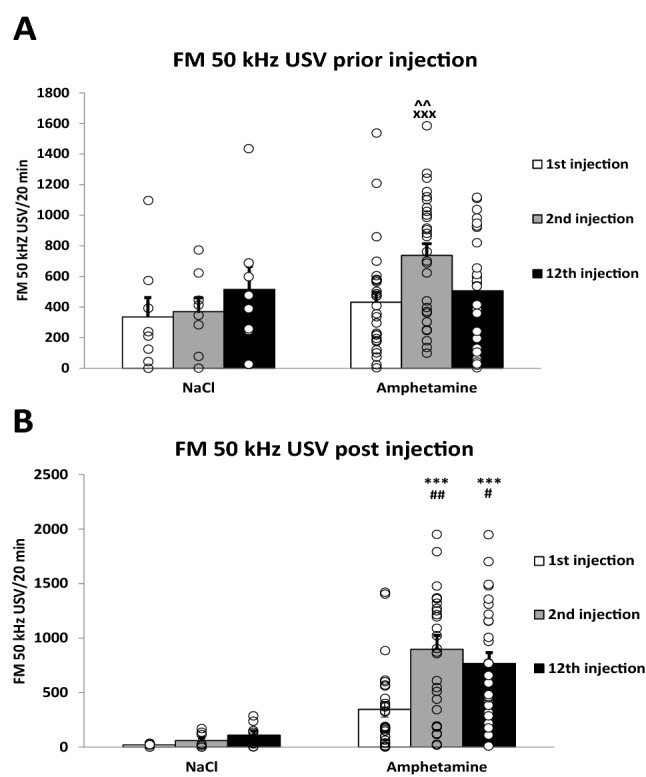
**A** The rate of FM 50-kHz USV during the 20-min sessions preceding the injection of 1st, 2nd, and 12th injection of Amph (test group, total *n* = 28) or NaCl (control group, *n* = 8); Two-way repeated measures ANOVA followed by post hoc Tukey’s test. ^xxx^*p* < 0.0001 vs the USV prior 1st Amph dose, ^^*p* < 0.001 vs the USV prior 12th Amph dose. **B** Effect of repeated Amph (test group, total *n* = 28) or NaCl (control group, *n* = 8) treatment on FM 50-kHz USV rate response after 1st, 2nd, and 12th injection; Two-way repeated measures ANOVA followed by post-hoc Tukey’s test. ^#^*p* < 0.05, ^##^*p* < 0.01 Amph vs. relevant NaCl group, *p 0.05, ***p < 0.001 vs. the USV post 1st Amph dose. The data are shown as the means + SEM. *FM 50-kHz USV* frequency-modulated ultrasonic vocalization, *Amph* amphetamine

FM 50-kHz USVs emitted during the time interval preceding administration of Amph (test group) or NaCl (ctrl) were considered in ctrl group rats as a response to the environment and procedure and in Amph-treated rats as a response to the Amph context. Two-way repeated measures ANOVA of square root-transformed data revealed no treatment effect (*F*_1,34_ = 1.51, *p* = 0.22) and showed a consecutive injections effect (*F*_2,68_ = 4.58 *p* = 0.014) and a treatment × consecutive injections interaction effect (*F*_2,68_ = 4.51 *p* = 0.015) (see Fig. 2A).

#### Amphetamine-induced FM 50-kHz USV rate

Two-way repeated measures ANOVA of square root-transformed 50 kHz USV data revealed a treatment effect (*F*_1,34_ = 23.62, *p* < 0.001), a consecutive injections effect (*F*_2,68_ = 10.84, *p* < 0.001) and a treatment × consecutive injections interaction effect (*F*_2,68_ = 3.17, *p* = 0.048) (see Fig. 2B).

### Effect of repeated amphetamine treatment on the tissue level of DA and its metabolites after various times since final amphetamine injection

**Fig. 3:**
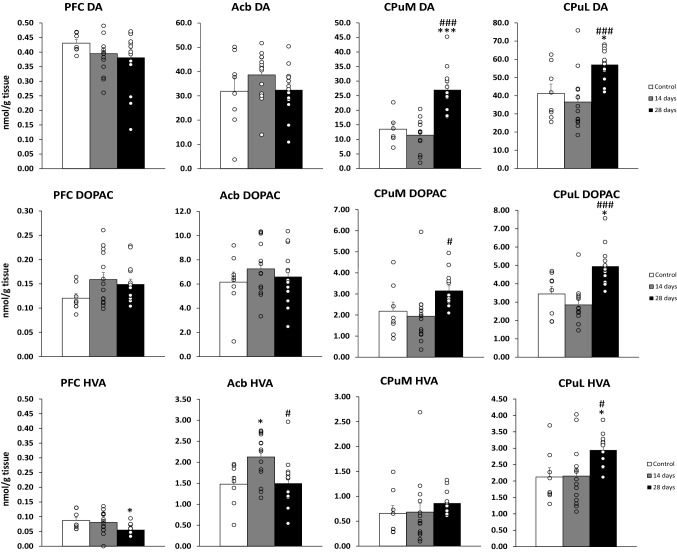
Tissue levels of DA, DOPAC, and HVA, in the rat PFC, Acb, CPuM, and CPuL after 12 injections of Amph (1.5 mg/kg) measured by HPLC 14 and 28 days following the last dose. Test groups received Amph with 14 (*n* = 14) or 28 (*n* = 14) days of withdrawal. The control group received NaCl (*n* = 8). One-way ANOVA followed by Post hoc Tukey’s test. *p < 0.05, ***p < 0.001 vs. the control group; ^#^*p* < 0.05, ^###^*p* < 0.001 vs. the group with 14 days withdrawal. The data are shown as the means + SEM. *DA* dopamine, *DOPAC* 3,4-dihydroxyphenylacetic acid, *HVA* homovanillic acid, *PFC* prefrontal cortex, *Acb* nucleus accumbens, *CPuM* dorsomedial striatum, *CPuL* dorsolateral striatum, *Amph* amphetamine

The changes in tissue levels of dopamine and its metabolites were compared in the ctrl group, 14 and 28 days after the last Amph dose (see Fig. 3).

### PFC

One-way ANOVA did not reveal any significant treatment effect for DOPAC (*F*_2,33_ = 2.02, *p* = 0.15) or DA (*F*_2,32_ = 0.75, *p* = 0.48), but showed a significant effect for HVA (*F*_2,33_ = 3.97, *p* = 0.029). Post-hoc Tukey’s test for unequal sample sizes showed a decrease in HVA levels 14 days after the last Amph dose compared to the ctrl group.

#### Acb

One-way ANOVA revealed a significant treatment effect for HVA (*F*_2,33_ = 5.68, *p* = 0.0076), and the post hoc Tukey's test for unequal sample sizes showed an increase in this metabolite 14 days after the last Amph dose compared to the ctrl and a decrease at 28 days after the last Amph dose compared to the level observed at 14 days. The same analysis showed no significant group effect for DOPAC (*F*_2,33_ = 0.67, *p* = 0.52) or DA (*F*_2,33_ = 1.35, *p* = 0.27).

#### CPuM

One-way ANOVA revealed a significant treatment effect for DOPAC (*F*_2,32_ = 4.12, *p* = 0.026) and DA (*F*_2,29_ = 21.57, *p* < 0.001) but no significant effect for HVA (*F*_2,32_ = 0.60, *p* = 0.55). As revealed by the post-hoc Tukey’s test for unequal sample sizes, DOPAC levels increased for the group with 28 days of abstinence compared to the group with 14 days of abstinence. The same test revealed that DA levels increased 28 days after the end of Amph administration compared to the ctrl and the group with 14 days of abstinence.

#### CPuL

One-way ANOVA revealed a significant treatment effect for DOPAC (*F*_2,32_ = 14.23, *p* < 0.001), DA (*F*_2,33_ = 9.76, *p* < 0.001), and HVA (*F*_2,33_ = 4.96, *p* = 0.013). The post-hoc Tukey’s test for unequal sample sizes showed an increase in DA and its metabolites (DOPAC, HVA) after 28 days of abstinence compared to the ctrl and 14-day withdrawal groups.

### Effect of repeated amphetamine treatment on tissue levels of 5-HT and 5-HIAA after various periods of abstinence

**Fig. 4:**
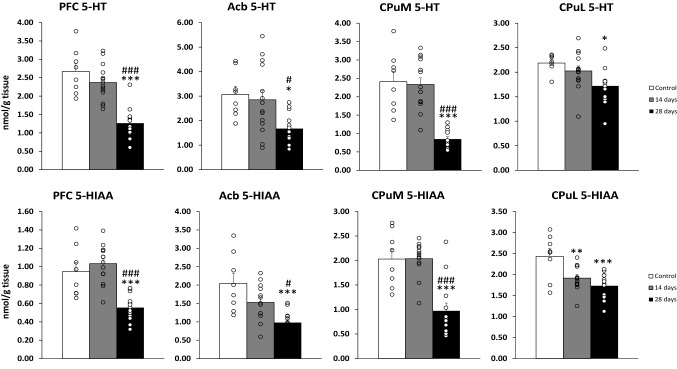
Tissue levels of 5-HT and 5-HIAA in the rat PFC, Acb, CPuM and CPuL after 12 injections of Amph (1.5 mg/kg) measured by HPLC 14 and 28 days following the last dose. Test groups received Amph with 14 (*n* = 14) or 28 (*n* = 14) days of withdrawal. The control group receiving NaCl (*n* = 8). One-way ANOVA followed by Post-hoc Tukey’s test. *p < 0.05, **p < 0.01, ***p < 0.001 vs. the control group; ^#^*p* < 0.05, ^###^*p* < 0.001 vs. the group with 14 days withdrawal. The data are shown as the means + SEM. *5-HT* serotonin, *5-HIAA* 5-hydroxyindoleacetic acid, *PFC* prefrontal cortex, *Acb* nucleus accumbens, *CPuM* dorsomedial striatum, *CPuL* dorsolateral striatum, *Amph* amphetamine

The changes in tissue levels of 5-HT and its metabolites were compared in the ctrl group, 14 and 28 days after the last Amph dose (see Fig. 4).

### PFC

One-way ANOVA showed a significant treatment effect for 5-HT (*F*_2,33_ = 27.42, *p* < 0.001) and 5-HIAA (*F*_2,33_ = 21.58, *p* < 0.001) in the prefrontal cortex. The post hoc Tukey’s test for unequal sample sizes showed a decrease in both 5-HT and 5-HIAA after 28 days of abstinence compared to the ctrl and the group with 14 days of abstinence.

#### Acb

One-way ANOVA showed a significant treatment effect for 5-HT (*F*_2,33_ = 6.24, *p* = 0.0050) and 5-HIAA (*F*_2,32_ = 11.49, *p* < 0.001). A decrease was observed for both 5-HT and 5-HIAA after 28 days of abstinence compared to the ctrl and the group with 14 days of abstinence, as confirmed by the post hoc test for unequal sample sizes.

#### CPuM

One-way ANOVA showed a significant treatment effect in 5-HT (*F*_2,31_ = 24.74, *p* < 0.001) and 5-HIAA (*F*_2,31_ = 18.49, *p* < 0.001). The post hoc Tukey's test for unequal sample sizes showed a decrease in 5-HT and 5-HIAA after 28 days of abstinence compared to the ctrl and the group with 14 days of abstinence.

#### CPuL

One-way ANOVA revealed a significant treatment effect for 5-HT (*F*_2,32_ = 5.24, *p* = 0.011) and 5-HIAA (*F*_2,32_ = 9.75, *p* < 0.001). The post hoc Tukey's test for unequal sample sizes showed a decrease in 5-HT levels after 28 days of abstinence compared to the ctrl and a decrease in 5-HIAA after both 14 and 28 days of abstinence compared to the ctrl.

## Discussion

Since relapses occur even after many years of abstinence, this may suggest that their cause is those of the drug-induced disorders that are characterized by persistence. The current study is focused on the changes in the tissue levels and interactions between DA and 5-HT after 14 and 28 days following the end of multiple Amph administrations.

### Behavior

The current study showed that repeated treatment with Amph-induced sensitization FM of 50-kHz USV rate. Our previous study showed that the rats exhibiting lower sensitivity to the rewarding effects of Amph self-administered more Amph [44] and the subsequent studies have shown that even a few doses of Amph induce long-term impairment of the DA system [45]. It seems interesting whether sensitivity to the rewarding effects of Amph is related to the persistence of the disturbances in DA and 5-HT levels after several weeks of the end of Amph administration. The doses used were shown to have a rewarding effect and the TIPS procedure induced sensitization. This procedure [35, 44], assumes that the first dose of the drug (day 1) induces sensitization, and the second dose (day 7) given after the time required for neurobiological changes to develop (6 days), reveals it [35]. Under this assumption, sensitization was assessed by the response to the second dose. It was higher than the response to the first dose. The administration of the following ten doses of Amph did not result in a further increase in FM 50 kHz USV, this indicates that, according to the TIPS procedure, the sensitization induced by the first dose was complete or that tolerance began to develop in parallel during subsequent doses. Here, unlike in previous studies [37], only a modest effect of the context of Amph administration on vocalization was observed; the anticipation of the USV before the second dose was higher than that before the start of Amph administration. However, it did not differ from the control levels (see Fig. 2A). This might be because the rats were housed singly. It can be assumed that following a prolonged period of social isolation a brief contact with a familiar friendly experimenter became rewarding, resulting in increasing baseline USV levels that were significantly higher than those observed in the group-housed rats and could mask the Amph-related context effect [41]. Vocalization was measured several weeks before the tissue was collected for neurochemical assays, so it was not directly related to the DA and 5HT levels but rather should be considered as an indicator of the individual sensitivity to the rewarding stimuli which, as previously shown, showed intra-individual stability [12]. Thus, FM 50 kHz USV before the first administration of Amph appears to determine sensitivity to environmental rewards. FM 50 kHz USV after injection of subsequent doses of Amph reflects the response to Amph reward, taking into account the factors such as the development of sensitization and tolerance. However, vocalization before the second and twelfth Amph doses may be influenced by the context of Amph administration and the aforementioned environmental factors, such as contact with the experimenter. No correlation was found between FM 50-kHz USV and levels of DA and 5HT, which suggested no relationship between the reward sensitivity and the ability of Amph to induce permanent changes in monoaminergic circuits.

### The DA system

The present study showed that repeated treatment with Amph-induced long-term changes in tissue levels of DA and its metabolites. After 14 days of abstinence, a transient increase in DA in Acb was observed, after 28 days an increase in DA in CPuM and DA, DOPAC and HVA in CPuL, and a decrease in HVA in PFC. Studies by others have shown that the DA response to psychostimulants in addicted, detoxified individuals is impaired [46–48]. Similar conclusions have been drawn from the rat studies by Kuczenski and Segal, who believe that chronic Amph administration leads to decreased DA synthesis and tissue levels of DA [44]. As mentioned earlier, most studies focus on the acute effects of psychostimulants on the DA system, while the studies assessing the effects of drugs after prolonged abstinence focus on cocaine or methamphetamine (METH) [49–51]. Although cocaine and METH are both psychostimulants, the differences in the mechanisms of action do not allow for close comparisons, and the studies that concern effects following long-term Amph withdrawal are few and inconsistent [44, 51, 52].

Of the brain regions studied, changes in levels of DA and its metabolites appeared primarily in the dorsal striatum, with similar changes in both the medial and lateral parts. This is interesting because, in recent years, there has been a growing interest in the role of the dorsal striatum in the development of habit, compulsion, and addiction [53–55]. Most researchers found a decrease in tissue DA level in this structure. Kuczenski and Segal [44] observed a decrease in tissue DA after 4 days since the end of Amph administration, as did Krasnova and colleagues [56] after 1, 7, and 14 days of abstinence. In the latter study, it was likely that large amounts of self-administered METH had an excitotoxic effect resulting in a decrease in levels of all tested amines (DA, 5-HT, norepinephrine) in all tested brain structures and all time points [56]. Also, postmortem studies on METH addicts reported significant decreases in striatal DA. However, these studies were performed shortly after the drug use, METH and Amph were still present in the blood of all subjects, so the authors suggested that this decrease was due to a massive DA depletion after METH use [57]. The results of our previous study [36] by microdialysis in the CPuL suggest that Amph induces a decrease in DA synthesis. In the current study, we expected that this phenomenon would be reflected by a decrease in DA tissue levels. However, after 14 days, unlike in the previous study after 11 days, only a nonsignificant decrease was observed. In contrast, a statistically significant increase in DA levels was found after 28 days which may indicate an increase in striatal DA synthesis at this time point. This effect is different from those reported in the studies cited above, but it should be noted that in those studies the time was shorter than 28 days. This increase may be a part of the process of extinction of Amph-induced disturbances, involving an alternating compensatory rise and a fall in DA levels.

### The 5-HT system

Repeated administration of Amph also has induced long-term changes in 5-HT system. All structures studied showed a decrease in tissue levels of 5-HT and 5-HIAA after 28 days of abstinence. Almost all psychostimulants with addictive properties are known to induce increased 5-HT activity in almost the whole brain, causing an increase in extracellular 5-HT release, which returns to normal after a few hours [58]. Fewer studies have focused on the changes in serotonin levels during abstinence, and the majority of these are related to cocaine. In cocaine-dependent humans, plasma 5-HT levels were found to increase after prolonged abstinence (more than 7 months) [59]. On the other hand, the studies in rats showed no differences in basal extracellular 5HIAA levels 3, 7, or 28 days after the last dose of Amph [60]. In our study, the changes in 5-HT and 5-HIAA in all structures examined were similar, with a strong decrease in the tissue levels after 28 days after the end of Amph administration. Toxic damage of 5-HT terminals may be one of the reasons for the reduced levels of 5-HT and 5-HIAA [61]. In this experiment, however, moderate doses of Amph were used, which did not induce effects such as for example body mass loss observed with excitotoxic doses [56] (see Table 1). The fact that increased tissue levels of DA and its metabolites are observed under these conditions also argues against this concept. It is believed that exposure to psychostimulants can alter 5-HT neuronal function over a long period and that serotonergic activity in the brain significantly alters reactivity to psychostimulants [62]. Pharmacological manipulations of 5-HT levels lead to changes in the response to cocaine. Many of the studies cited in the discussion are on cocaine, as those on Amph are few. Although the action of both psychostimulants leads to increased DA and 5-HT levels, this is achieved in different ways. Cocaine acts by blocking transporters’ reuptake from the synaptic cleft, while Amph acts as a substrate for these transporters and competes with them for binding. By diffusion exchange, Amph is transported into the cell, while transmitters are carried outside the cell. Thus, it is important to keep in mind that the effects exerted by these two stimulants on DA, 5-HT and the interactions between them may be different [63].

The increased 5-HT availability induced by the administration of the amino acid precursor of 5-HT, tryptophan, reduced the locomotor response to cocaine [64]. In contrast, in cocaine-dependent humans Cox and co-workers, using [11C] raclopride PET showed that a decrease in tryptophan levels provoked by oral administration of a tryptophan-deficient amino acid mixture augmented drug-seeking and striatal DA release in responses to cocaine [65]. The decrease in tissue levels of 5-HT and its metabolite 5-HIAA in the striatum observed after 28 days of abstinence may result in craving incubation [66]. The aforementioned effect is associated with a progressive increase of drug craving during abstinence, which in cocaine self-administered rats reaches a maximum after several weeks of abstinence from cocaine [67] or METH [68]. The severity of the craving is an important parameter as it facilitates the prediction of treatment outcomes [66].

### Correlations of amines with their metabolites

Since in the Acb and both parts of the striatum studied, DA was correlated with its metabolite DOPAC in a similar manner in the ctrl and in rats that received Amph, it appears that the perturbation caused by Amph administration does not involve DA metabolism. For 5-HT and its metabolite 5-HIAA, correlations were observed in all groups in both parts of the striatum. However, in the Acb, the correlations occurred only 14 and 28 days after the last Amph dose, which may suggest an effect of Amph on 5-HT metabolism in this structure.

### DA and 5-HT interactions

Because there is a functional and anatomical relationship between the DA and 5-HT systems, with both being involved in the response to psychostimulants and playing an important role in the development of addiction [69], it is important to analyze the relations between these systems within and between the brain structures studied.

**Fig. 5:**
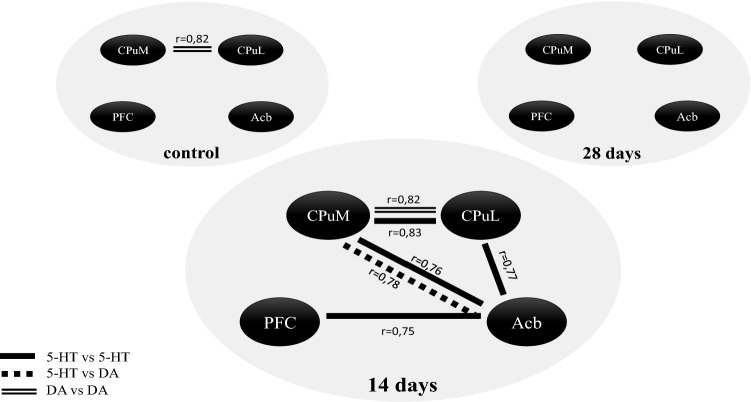
Pearson’s correlations with Bonferroni–Holm correction between DA and 5-HT levels measured by HPLC, in the control group (*n* = 8) and at 14 (*n* = 14) and 28 (*n* = 14) days after completion of 12 doses of Amph (1.5 mg/kg). *p* < 0.05 in all cases. *5-HT* serotonin, *DA* dopamine, *Amph* amphetamine

As shown in Fig. 5, the intra- and inter-structural correlations between 5-HT and DA appear primarily after 14 days of abstinence. At this time, tissue levels of amines and their metabolites were not significantly different from those in the ctrl. However, this precedes the increases in DA (in both parts of the striatum) and the decreases in 5-HT (in all structures studied) observed after next 14 days (28 days after the last Amph dose). The fact that so many correlations, which have not been present in the ctrl, appear only after 14 days after completion of Amph administration indicates that Amph has triggered processes that involve both of these systems and affect the relationship between them in the brain areas studied. It resembles, to some extent, the effect of some psychotropic drugs, whose clinical effect is most noticeable after a few weeks [70].

5-HT and DA play important and interacting roles in regulating mood, impulsive actions, and risky decision-making [71, 72]. Human and animal studies in which 5-HT levels have been manipulated strongly support a role for serotonin in affective processing. Lesion of 5-HT neurons increased the activity of DA neurons [73], but an increase in 5-HT tone inhibits dopamine transmission [74]. However, the direction of association and specificity for aversive or rewarding events remain controversial. Explaining the nature of the interaction between 5-HT and DA is difficult, as both inhibitory and excitatory roles for 5-HT have been suggested, which may be due to the different distributions and functional roles of 5-HT receptor subtypes in dopaminergic circuits. Disorders of 5-HT transmission often have opposite effects to those of DA disorders on impulsive–compulsive behavior, probably because the two monoamine systems are mutually regulated, often through antagonistic interactions [75].

**Fig. 6:**
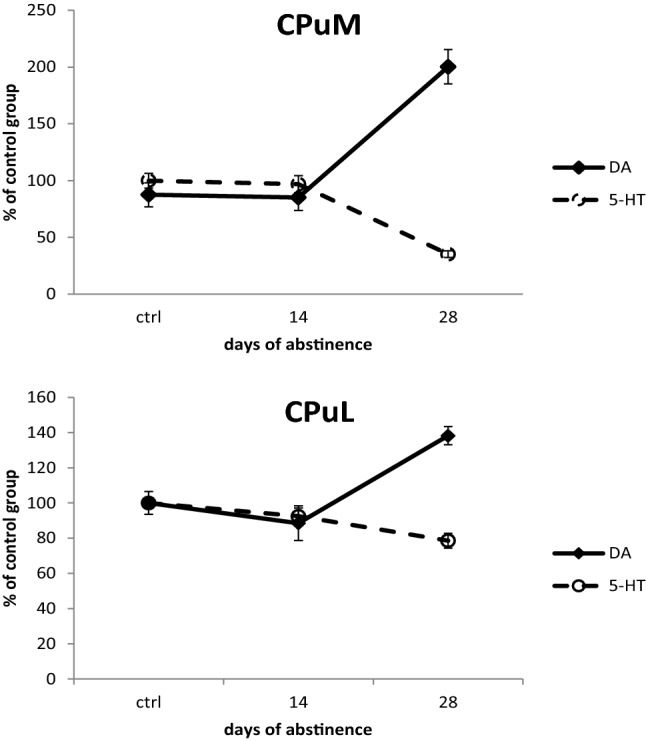
Comparison of 5-HT and DA changes in CPuM and CPuL 14 (*n* = 14) and 28 (*n* = 14) days after administration of 12 injections of Amph (1.5 mg/kg) measured by HPLC. *5-HT* serotonin, *DA* dopamine, *CPuM* dorsomedial striatum, *CPuL* dorsolateral striatum, *Amph* amphetamine

The additive effect of serotonin and DA conveys important reward-related information and is subjectively euphoric, while its dissociation may be associated with aversive feelings [76] connected to a negative emotional state and the formation of craving during withdrawal. In our study, opposing changes in tissue levels of DA and 5-HT were particularly pronounced in the dorsal striatum (see Fig. 6). Perhaps associated with this is the emergence of a negative emotional state and craving during withdrawal, which can promote relapse.

In summary, it is known that drug-induced neurobiological disturbances normalize over time in most individuals. However, in some cases, brain functions remain permanently disturbed which can lead to the development of addiction. Therefore, it is crucial to look for long-term drug effects. The results of our experiment showed that the impairment in DA and 5-HT systems induced by Amph persists long after the drug has been eliminated from the body and its observable effects have disappeared. The appearance of numerous intra- and inter-structural correlations between DA and 5-HT 14 days after the end of Amph administration may indicate the existence of drug-induced processes in which serotonergic and dopaminergic neurotransmission interact. The strongest changes in monoamine levels (increase in DA and decrease in 5-HT) were observed after 28 days of abstinence. This indicates that Amph induces processes that need some time to reveal their effects. The opposite direction of changes in DA and 5-HT levels may be related to a negative emotional state of the animals, which may contribute to the incubation of cravings and consequently to the promotion of relapse. Determining which of the observed sustained changes may be associated with the emergence of addiction requires further studies on large cohorts, since the percentage of addicts that manifest these characteristics in the group of drug users is relatively small.

## Data Availability

Data are available from the corresponding author on reasonable request.

## Abbreviations

Acb: Nucleus accumbens
Amph: Amphetamine
CPuM: Dorsomedial striatum
CPuL: Dorsolateral striatum
ctrl: Control group
DA: Dopamine
DOPAC: 3,4-Dihydroxyphenylacetic acid
FM 50-kHz USV: Frequency-modulated ultrasonic vocalization
HPLC: High-pressure liquid chromatography
HVA: Homovanillic acid
*ip*: Intraperitoneal injections
METH: Methamphetamine
PFC: Prefrontal cortex
TIPS: Two Injection Protocol of Sensitization
USV: Ultrasonic vocalization
5-HIAA: 5-Hydroxyindoleacetic acid
5-HT: Serotonin
